# The role and implications of left atrial fibrosis in surgical mitral valve repair as assessed by CMR: the ALIVE study design and rationale

**DOI:** 10.3389/fcvm.2023.1166703

**Published:** 2023-05-12

**Authors:** Sulayman el Mathari, Jolanda Kluin, Luuk H. G. A. Hopman, Pranav Bhagirath, Maurice A. P. Oudeman, Alexander B. A. Vonk, Aart J. Nederveen, Susanne Eberl, Robert J. M. Klautz, Steven A. J. Chamuleau, Pim van Ooij, Marco J. W. Götte

**Affiliations:** ^1^ Department of Cardiothoracic Surgery, Amsterdam University Medical Center, Amsterdam, Netherlands; ^2^ Department of Cardiothoracic Surgery, Erasmus University Medical Center, Rotterdam, Netherlands; ^3^ Department of Cardiology, Amsterdam University Medical Center, Amsterdam, Netherlands; ^4^ Department of Radiology and Nuclear Medicine, Amsterdam University Medical Center, Amsterdam, Netherlands; ^5^ Department of Anesthesiology, Amsterdam University Medical Center, Amsterdam, Netherlands; ^6^ Department of Cardiothoracic Surgery, Leiden University Medical Center, Rotterdam, Netherlands

**Keywords:** left atrial fibrosis, atrial remodeling, mitral valve repair surgery, mitral regurgitation, cardiac magnetic resonance imaging, ALIVE trial

## Abstract

**Background:**

Patients with mitral regurgitation (MR) commonly suffer from left atrial (LA) remodeling. LA fibrosis is considered to be a key player in the LA remodeling process, as observed in atrial fibrillation (AF) patients. Literature on the presence and extent of LA fibrosis in MR patients however, is scarce and its clinical implications remain unknown. Therefore, the ALIVE trial was designed to investigate the presence of LA remodeling including LA fibrosis in MR patients prior to and after mitral valve repair (MVR) surgery.

**Methods:**

The ALIVE trial is a single center, prospective pilot study investigating LA fibrosis in patients suffering from MR in the absence of AF (identifier NCT05345730). In total, 20 participants will undergo a CMR scan including 3D late gadolinium enhancement (LGE) imaging 2 week prior to MVR surgery and at 3 months follow-up. The primary objective of the ALIVE trial is to assess the extent and geometric distribution of LA fibrosis in MR patients and to determine effects of MVR surgery on reversed atrial remodelling.

**Implications:**

This study will provide novel insights into the pathophysiological mechanism of fibrotic and volumetric atrial (reversed) remodeling in MR patients undergoing MVR surgery. Our results may contribute to improved clinical decision making and patient-specific treatment strategies in patients suffering from MR.

## Introduction

**Figure 1:**
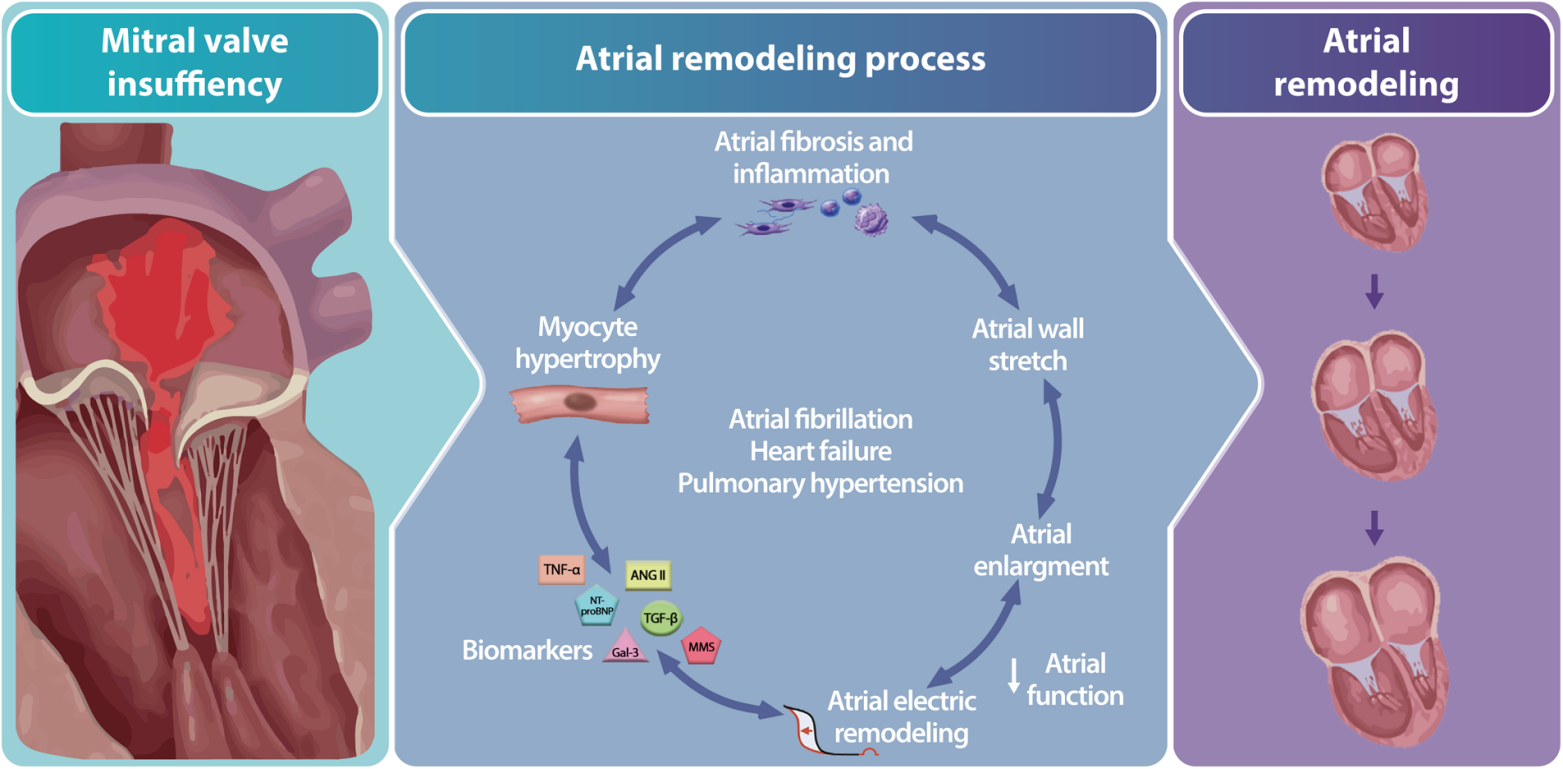
Illustration of the atrial remodeling process due to mitral valve regurgitation including associated drivers. Mitral valve regurgitation triggers atrial remodeling by activating neurohumoral mediation reflected by a change in biomarker levels resulting in myocyte hypertrophy, atrial fibrosis and inflammation, atrial wall stretch, enlargement and electrical remodeling. *Abbreviations: ANG II; angiotensin II, Gal-3; galectin-3, MMS; matrix metalloproteinases NT-proBNP; N-terminal pro-brain natriuremic peptide, TGF-β; growth factor beta, TNF-*α*; tumor necrosis factor-alpha*.

Patients with primary mitral regurgitation (MR) frequently suffer from left atrial (LA) remodeling, caused by volume overload and subsequent atrial dilation (1). The associated myocardial stretch, increased wall tension and involved neurohumoral modulators including transforming growth factor beta (TGF-*β*), tumor necrosis factor-alpha (TNF-α), angiotensin II (Ang II), matrix metalloproteinases (MMPs), galectin-3 (Gal-3) and N-terminal pro-brain natriuremic peptide (NT-proBNP), trigger a cascade of pathways that ultimately may lead to formation of atrial fibrosis as part of the atrial remodeling process (Figure 1) (2–7).

In different categories of patients without primary MR, it has been demonstrated that the presence of atrial fibrosis is associated with an increased risk of adverse events including atrial fibrillation (AF), stroke, heart failure, pulmonary hypertension, reduced quality of life and eventually a shorter life expectancy (8–11). In addition, presence and amount of LA fibrosis was found to be a strong predictor for arrhythmic treatment efficacy and long-term outcome in patients suffering from AF (12, 13). Several studies have suggested that LA fibrosis may also be present in MR patients, but its role and consequences remain yet to be determined (1, 2, 11, 14–17).

In the last decade, high spatial resolution 3-dimensional (3D) late gadolinium enhanced (LGE) cardiac magnetic resonance (CMR) imaging has emerged as a noninvasive tool for the visualization and quantification of LA fibrosis (18–20). Extent and geometric distribution of LA fibrosis can be assessed using advanced post-processing tools, allowing to study LA fibrosis in primary MR patients on a routine base. This may be important, since presence and distribution of LA fibrosis in primary MR patients may have clinical implications for patient-specific treatment strategies.

In current clinical practice, mitral valve repair (MVR) surgery is the ultimate treatment for primary MR (21, 22). Because progression of LA remodeling due to prolonged presence of MR is associated with worse outcomes, including increased cardiovascular morbidity and mortality (23–26), timing of surgical intervention is crucial to avoid severe LA remodeling.

Currently, the indication and timing for MVR in these patients is mainly based on severity of MR and the presence of symptoms or left ventricular dysfunction (end systolic dimension ≥ 4.0 cm or left ventricular ejection fraction ≤ 60%) (27). Yet, despite these long-standing guidelines on the indication and timing for MVR surgery in severe primary MR, 20% of the patients present postoperative with a reduced left ventricular ejection fraction (LVEF) and an increased risk for heart failure (28). This argues for early surgery in asymptomatic patients at low risk for postoperative adverse events. Such an approach demands additional parameters to support the choice for either early surgery or justified watchful waiting.

For this reason, guidelines suggest to take LA remodeling into account for clinical assessment and therapy stratification (27). Since atrial fibrosis is considered to be part of the LA remodeling process, it may be clinically relevant to explore the role of LA fibrosis in primary MR patients (29, 30). Also pathophysiologic data of LA reversed remodeling after MVR remain largely unexplored (31), and data about reversal or expansion of LA fibrosis is lacking.

Therefore, in this study, we combine advanced cardiac MRI and post-processing techniques prior to and after MVR surgery, to study LA fibrosis in these patients. It is hypothesized that LA fibrosis in primary MR patients may be present, and that the LA fibrotic surface area may paradoxically increase after MVR surgery due to a reduction in global LA volume (i.e., reversed remodeling) and total surface area, while the fibrotic area remains unaffected.

## Methods and analysis

The ALIVE trial is a prospective single center pilot study applying advanced CMR imaging and post-processing in primary MR patients undergoing MVR surgery. This study will be executed at the Amsterdam University Medical Centers (AmsterdamUMC) in compliance with the Declaration of Helsinki and in accordance with the Medical Research Involving Human Subjects Act (WMO). The study has been approved by the institutional ethics committee on human research under registration number 2021_201—NL78497.018.21 (December 14th, 2021).

### Patient enrollment

Patients with chronic severe primary MR and without AF, who meet criteria for MVR surgery (32) will be included. After informed consent is obtained, patients will be enrolled in the study. Study selection criteria are listed in Table 1.

**Table 1:** Inclusion and exclusion criteria.

**Inclusion criteria**	Age > 18 years
	Elective surgical candidate
	Meeting criteria for MVR surgery according to clinical guidelines (class I recommendation) (32) • Symptomatic chronic severe mitral regurgitation owing to degenerative valve disease with a left ventricular ejection fraction (LVEF) > 30%. • Asymptomatic chronic severe mitral regurgitation owing to degenerative valve disease with a LVEF <60% and/or a left ventricular end-systolic diameter (LVESD) > 45 mm.
	Signed informed consent
**Exclusion criteria**	Age > 80 years
	History of cardiac surgery
	History of AF
	Extensive comorbidities besides MR (i.e., cancer, other chronic diseases)
	Known allergic reaction to contrast agent gadolinium
	Contra-indications for CMR with contrast administration (e.g., severe claustrophobia, metal implants, severe renal failure resembling <30 eGFR, severe asthma and known hypersensitivity for gadolinium)

AF; atrial fibrillation, MR; mitral regurgitation, MVR; mitral valve repair surgery, LVEF; left ventricular ejection fraction, LVESD; left ventricular end-systolic diameter, CMR; cardiac magnetic resonance.

A matched control group, including patients without MR and no AF, will be collected from an existing CMR imaging database of the Amsterdam UMC. These control patients underwent the same scan protocol, for a different indication. Preoperative results of the primary MR study population will be compared to control data to assess baseline differences in LA fibrosis and other LA remodeling parameters between MR and non-MR patients.

### Study protocol

**Figure 2:**
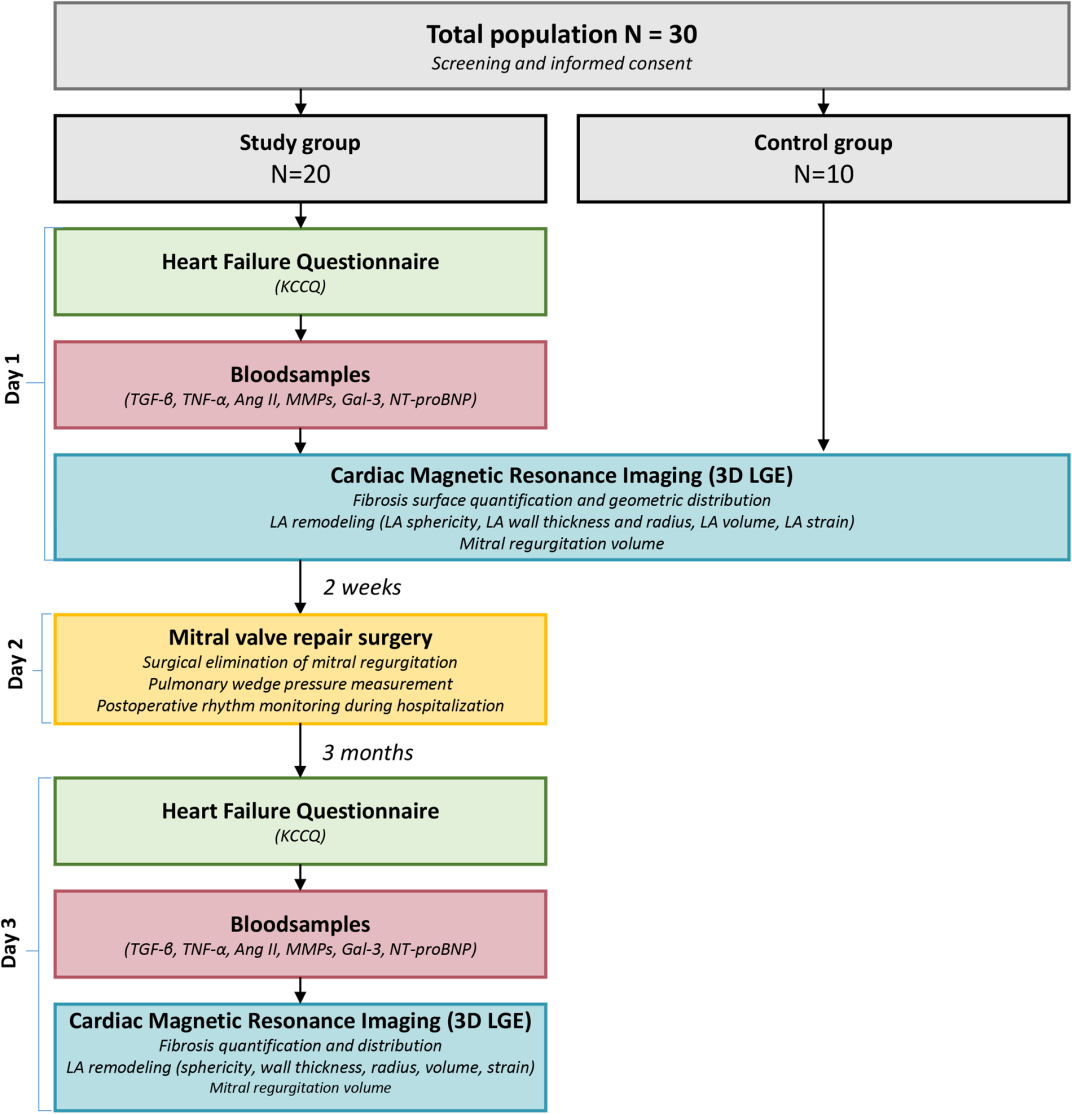
Flow chart representing the study design of the ALIVE study. Effective study time includes 3 days. Day 1 consists of preoperative 3D LGE CMR imaging, taking blood samples and a KCCQ questionnaire. Day 2 involves MVR surgery. The last study day is a repetition of the first day. Time between the study days is respectively two weeks and three months. *Abbreviations: ANG II; angiotensin II, Gal-3; galectin-3, MMS; matrix metalloproteinases, NT-proBNP; brain N-terminal pro-natriuretic peptide, KCCQ; Kansas City cardiomyopathy questionnaire, LA; left atrium, LGE; late gadolinium enhancement, TNF-*α*; tumor necrosis factor-alpha*.

The study protocol is shown in Figure 2. On day 1, primary MR patients will undergo a CMR scan including 3D LGE imaging to determine presence, localization and amount of LA fibrosis., blood samples will be collected to analyze levels of associated neurohumoral modulators (TGF-*β*, TNF- *α*, Ang II, MMPs, Gal-3, NT-proBNP) that contribute to LA fibrosis development. In addition, the Kansas City Cardiomyopathy Questionnaire (KCCQ) (33) will be conducted to assess severity of cardiac complaints. Two weeks after the CMR scan, patients will undergo MVR surgery. Prior to the surgical incision, while the patient is under anesthesia, a Swan-Ganz catheter will be introduced to measure pulmonary Wedge pressure. The measurement of the pulmonary Wedge pressure is considered a surrogate for the LA pressure.

Postoperatively, the heart rhythm of all patients will be monitored until hospital discharge and all available ECGs and holter investigations during follow-up will be reviewed for any arrhythmia. Three months after surgery, patients will undergo follow-up CMR imaging to assess particularly postoperative LA reversed-remodeling changes including fibrosis, but also cardiac function parameters. Also, blood sample testing for associated neurohumoral modulators and a KCCQ questionnaire will be repeated during this follow-up visit. Finally, also an ECG will be obtained during this same visit.

#### CMR

**Figure 3:**
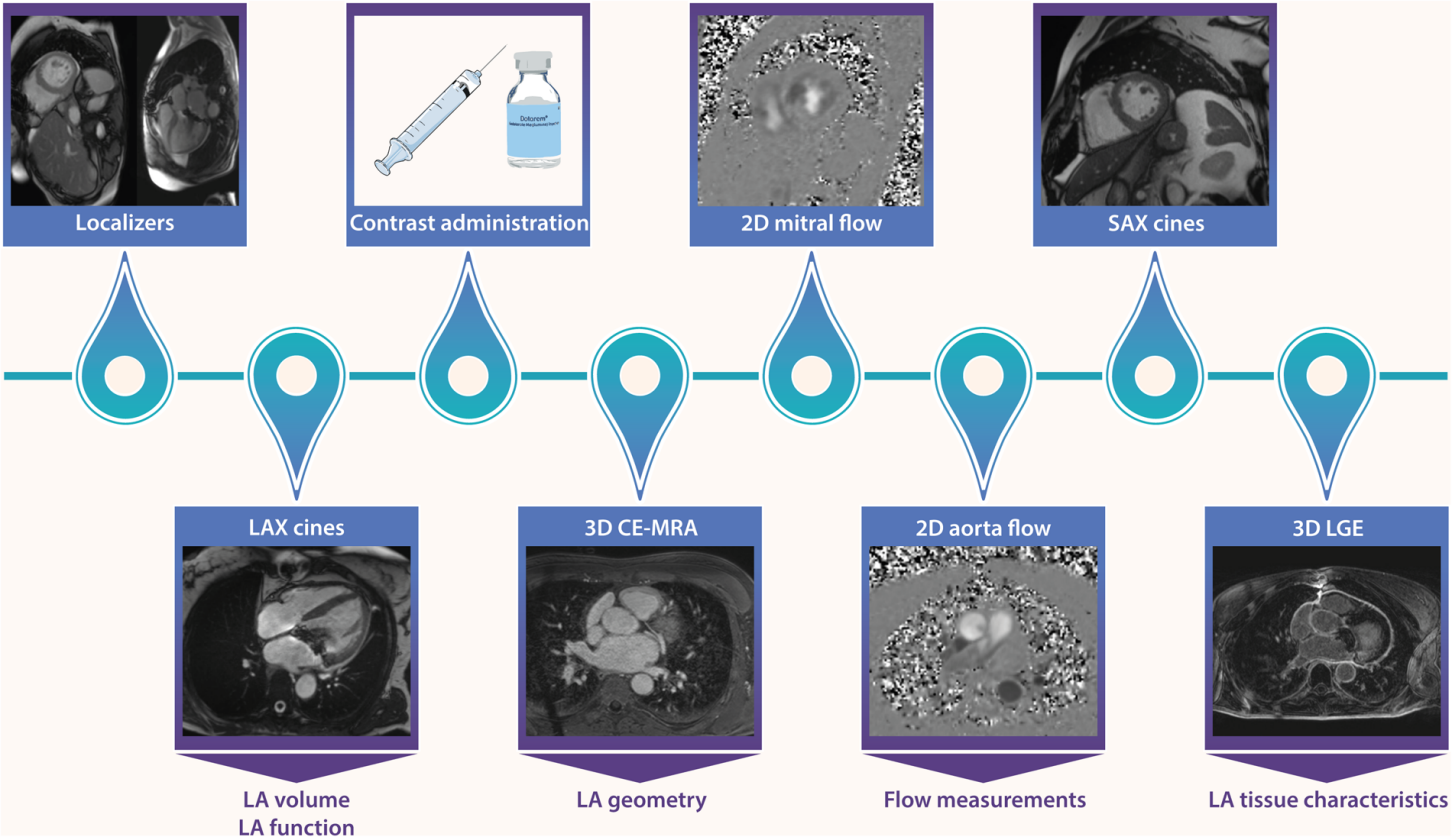
CMR scan protocol of the ALIVE trial. Cine imaging is performed in long axis (LAX) orientation. After contrast administration ((Dotarem®, Guerbet, Roissy, France), a 3D contrast-enhanced MR angiogram (CE-MRA) of the left atrium and pulmonary veins will be obtained immediately after a single dose bolus injection. Two-dimensional flow measurements will be performed to quantify severity of MR and a stack of short axis (SAX) is acquired. Finally, high resolution 3D LGE images will be acquired using a navigator-based respiration and ECG-gated inversion recovery prepared gradient echo pulse sequence, which is applied 17 min after contrast injection to limit variation between scans. *Abbreviations: LAX; long axis, LGE; late gadolinium enhancement, MRA; Magnetic resonance angiography, SAX; Short axis.*

Scans are performed using a 1.5 Tesla CMR system with 32-channel coil (Sola scanner, Siemens Erlangen, Germany). The scan protocol includes balanced steady state free precision (SSFP) cine imaging in long axis orientation (two-chamber view, four-chamber view) and a stack of short axis. Two-dimensional through-plane flow measurements at the aortic and mitral valve will be performed to quantify severity of MR. An ECG-gated free-breathing 3D contrast-enhanced MR angiogram (CE-MRA) of the LA and pulmonary veins will be obtained immediately after a 20 ml (1 ml/sec) single dose bolus injection of contrast agent (Dotarem®, Guerbet, Roissy, France) followed by a body weight dependent slow infusion of contrast agent (slow infusion dose; 2.5–30.0 ml, infusion rate; 0.1–0.25 ml/sec) equal to a total dose of 0.4 ml/kg. Finally, high resolution 3D LGE images will be acquired using a navigator-based respiration and ECG-gated inversion recovery prepared gradient echo pulse sequence, which is applied approximately 17 min after contrast injection. Acquisition parameters for the 3D LGE acquisition are as follows; repetition time 5.5 ms; echo time 3.0 ms; flip angle 25°; in-plane resolution 1.25 × 1.25 mm; slice thickness 2.5 mm (reconstructed to 0.625 × 0.625 × 1.25) (18). Figure 3 shows an overview of the imaging protocol.

#### Left atrial remodeling

LA remodeling will be assessed by measuring LA volume and LA geometry. LA volume (ml) is calculated from two- and four-chamber cine images using the biplanar method. Accordingly LA volumes are divided by body surface area (BSA) to calculate LA volume index (LAVI).

#### Left atrial geometry and wall tension

**Figure 4:**
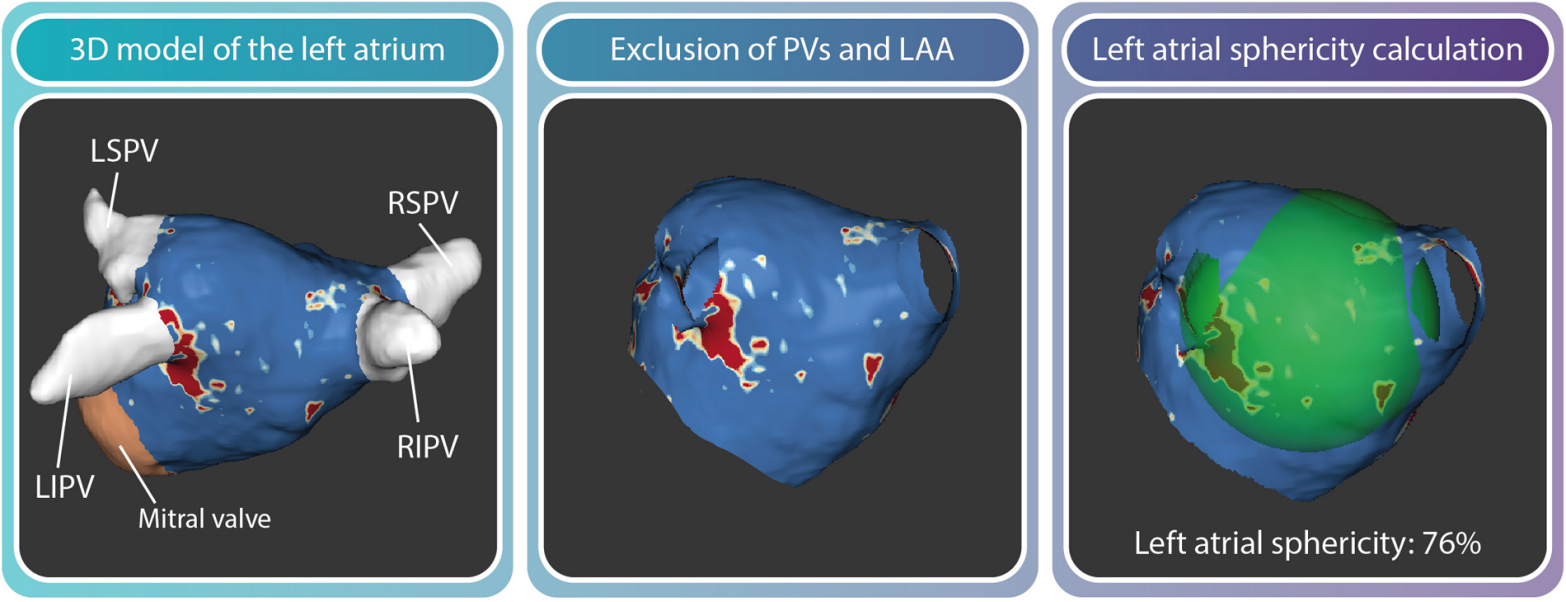
A 3D reconstruction of the LA can be made using dedicated segmentation software. After the pulmonary veins and LA appendage are excluded, LA sphericity is calculated automatically. The center of LA mass is determined and the average radius between all points of the LA wall and the center of mass is calculated in percentages.

Assessment of LA geometry will be performed by calculating the LA sphericity. This parameter is defined as a measure of agreement between the LA shape and a perfect sphere (Figure 4). In case of LA volume overload, increased spherical remodeling may occur as a result of geometrical adaptation to the increased volume and associated increased wall stress (34).

LA sphericity is quantified by using open source software ADAS 3D (Galgo Medical, Barcelona, Spain). A 3D reconstruction of the LA is semi-automatically segmented from the 3D CE-MRA images. The LA appendage and pulmonary veins are excluded from the total LA 3D reconstruction leaving a 3D LA shell of which the LA sphericity is calculated using a previously published algorithm (34). Outcome measures are defined in percentages, where 100% represents a perfect sphere and non-spherical shapes have lower % values.

To calculate LA wall tension, we apply Laplace's law, which relates the tension in the wall of a spherical chamber to the intraluminal pressure and radius (T = *p* • r/2 T). In this case, we take into account the LA sphericity, wall thickness, and pulmonary Wedge pressure derived from the Swan-Ganz measurement. Since the LA does not have a perfect spherical shape, the atrial wall thickness may vary locally, while Wedge pressure is a surrogate for LA pressure, it is obvious this is a simplification of reality. Nevertheless, we consider this is currently the highest achievable for integrated analysis of atrial shape, function and tissue characteristics.

#### Left atrial function

LA strain and ejection fraction analysis will be performed using dedicated post-processing software (Circle Cardiovascular Imaging, Inc, Calgary, Canada). LA ejection fraction will be calculated by using LA minimal and maximal volume. For strain analysis, LA endocardial and epicardial borders will be traced in the end-diastolic phase of two- and four-chamber cine images and subsequently tracked over the cardiac cycle. Measurements will be divided into LA reservoir, conduit and contractile strain.

#### Left atrial fibrosis

To assess LA fibrosis, 3D LGE images will be analyzed using commercially available post-processing software ADAS 3D (Galgo Medical, Barcelona, Spain). The 3D LGE images will undergo stringent quality control (i.e., artefacts, proper myocardial nulling) by two experienced and level III certified CMR readers prior to post-processing. Images will be excluded from analysis if quality is deemed insufficient.

**Figure 5:**
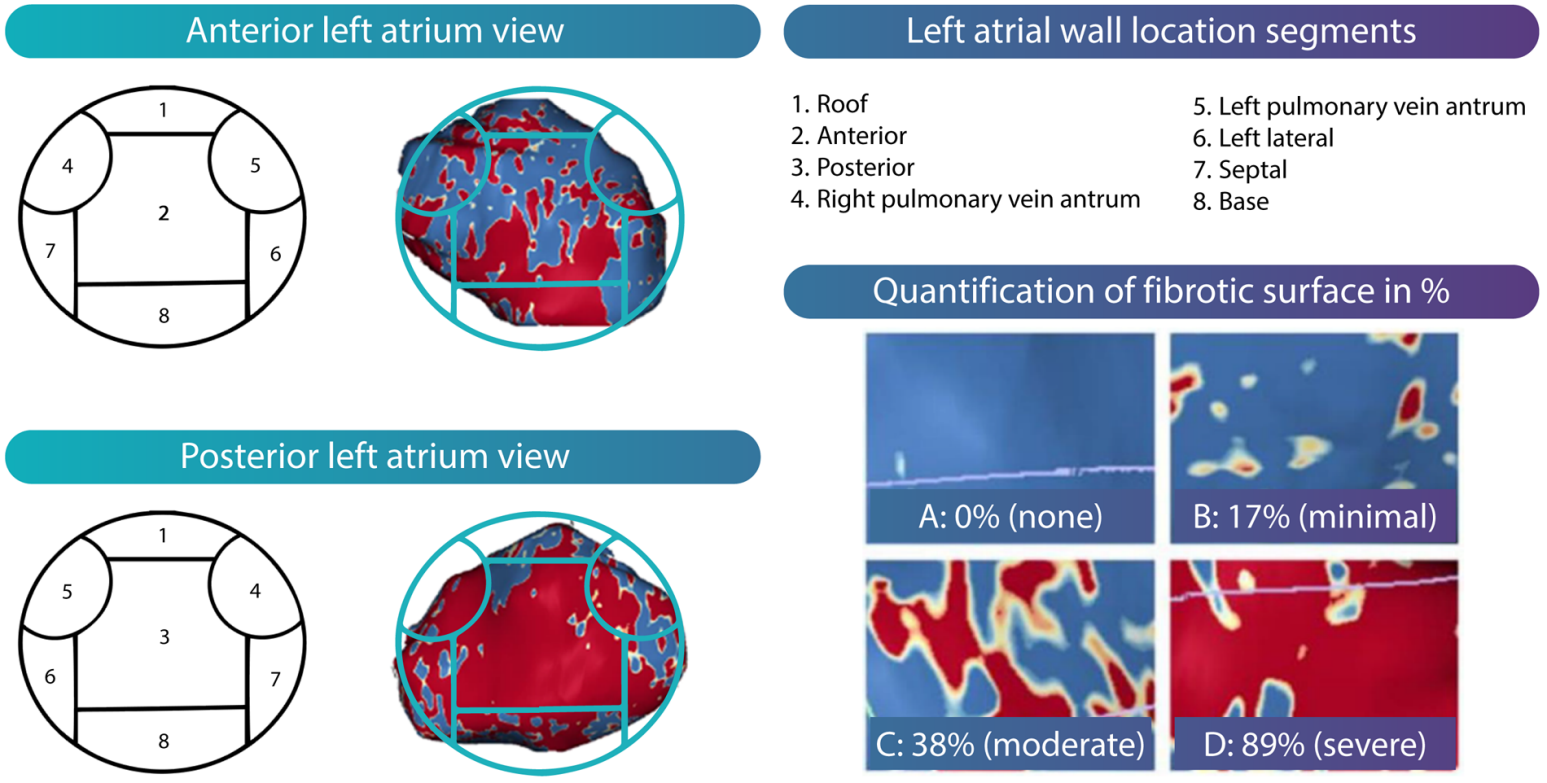
Segmental analysis of LA fibrosis. Different atrial segments are demonstrated both in the anterior and posterior atrium view. Examples of regional distribution of LA fibrosis is given in both views. Quantification of fibrotic surface area in percentages is done automatically for every segment.

Quantification of LA fibrosis surface and its geometric distribution will be assessed using LA 3D reconstructions. These reconstructions are obtained by semi-automated segmentation in multiple axial planes by drawing mid-atrial wall contours on the 3D LGE images, followed by automatic interpolation of the contours to the intermediate slices. Before fibrosis calculation, the LA appendage and pulmonary veins are excluded at their ostia defined as the point of deflection from the LA wall. A default image intensity ratio threshold of 1.2 (1.2 times mean blood pool) is used to calculate the presence and amount of LA fibrosis (35). Figure 5 shows a typical example of regional fibrosis analysis. The LA wall is divided into 8 segments to determine geometric distribution of fibrosis. The extent of fibrosis is calculated automatically as percentage of quantified surface area.

#### Surgical technique

Surgical MVR is the recommended therapy for patients who require treatment for primary MR (27). This involves the use of a sternotomy approach, which includes mitral annuloplasty and may also involve the implantation of artificial neo-chordae or valve tissue resection in cases where there is excessive valve tissue (36). Multiple studies have reported favorable outcomes for this approach, indicating its effectiveness and safety for this specific patient group (21, 22, 37).

#### Neurohumoral modulators associated with left atrial structural remodeling

Since biomarker plasma levels are known to increase with progression of disease (38, 39), blood samples will be collected two weeks before and 3 months after surgery to assess neurohumoral modulators associated with LA structural remodeling (TGF-*β*, TNF- *α*, Ang II, MMPs, Gal-3, NT-proBNP). Samples are drawn into Ethylene Diamine Tetra Acetic acid (EDTA), serum and non-additive tubes. The blood samples are separated with centrifugation at a speed of 4,000 rotations per minute for 20 min whereafter the plasma of each tube is isolated into smaller sample tubes for storage at minus 80 Celsius degree. After collection of all samples, Enzyme Immunoassays (EIA) and Enzyme-Linked ImmunoSorbent Assays (ELISA) will be performed to analyze levels of each of the earlier mentioned neurohumoral modulators.

### Outcome measures

The primary objective of the ALIVE study is to study the effects of (reduced) volume overload on the LA wall texture (presence, amount and geometric distribution of atrial fibrosis) in primary MR patients, prior to and after elective MVR surgery.

Secondary objectives are to assess 1) the impact of LA fibrosis on myocyte contractile function (atrial strain) and 2) the impact of LA remodeling on right atrial function. In addition, to gain insight in the relevance of LA fibrosis in relation to stratification and timing of MVR surgery, 3) the relation between the presence of LA fibrosis prior to MVR surgery and short-term clinical outcome (i.e., procedural complications, duration of hospital stay, post-operative AF, and heart failure) will be evaluated.

### Sample size calculation and data analysis

As no previous studies have investigated the potential presence of LA fibrosis using CMR in this specific patient population, there is a lack of literature available to guide a valid sample size calculation. As an initial assessment, we have decided to include 20 patients with primary mitral regurgitation who are undergoing MVR surgery. While we acknowledge that this sample size may be considered small, our pilot study aims to provide important exploratory data regarding the presence and extent of LA fibrosis in patients with primary MR.Presence, amount and geometric distribution of LA fibrosis will be quantified and a comparison will be made between the preoperative and postoperative state. Quantified measures will be corrected for multiple testing using the Bonferroni method. Based on the distribution of continuous outcomes, independent samples *T*-test or Mann-Whitney *U* test are used as appropriate.

## Pilot data

As pilot data, we present a 59 years old female with symptomatic severe primary MR, meeting the guideline criteria for MVR surgery. This patient underwent a preoperative CMR scan according to the above outlined protocol. Two weeks later, surgical MVR was successfully performed with MV annuloplasty and implantation of artificial neo-chordae. Further hospitalization was uncomplicated and the patient was discharged 7 days after surgery. A postoperative CMR scan was acquired 3 months after discharge.

**Figure 6:**
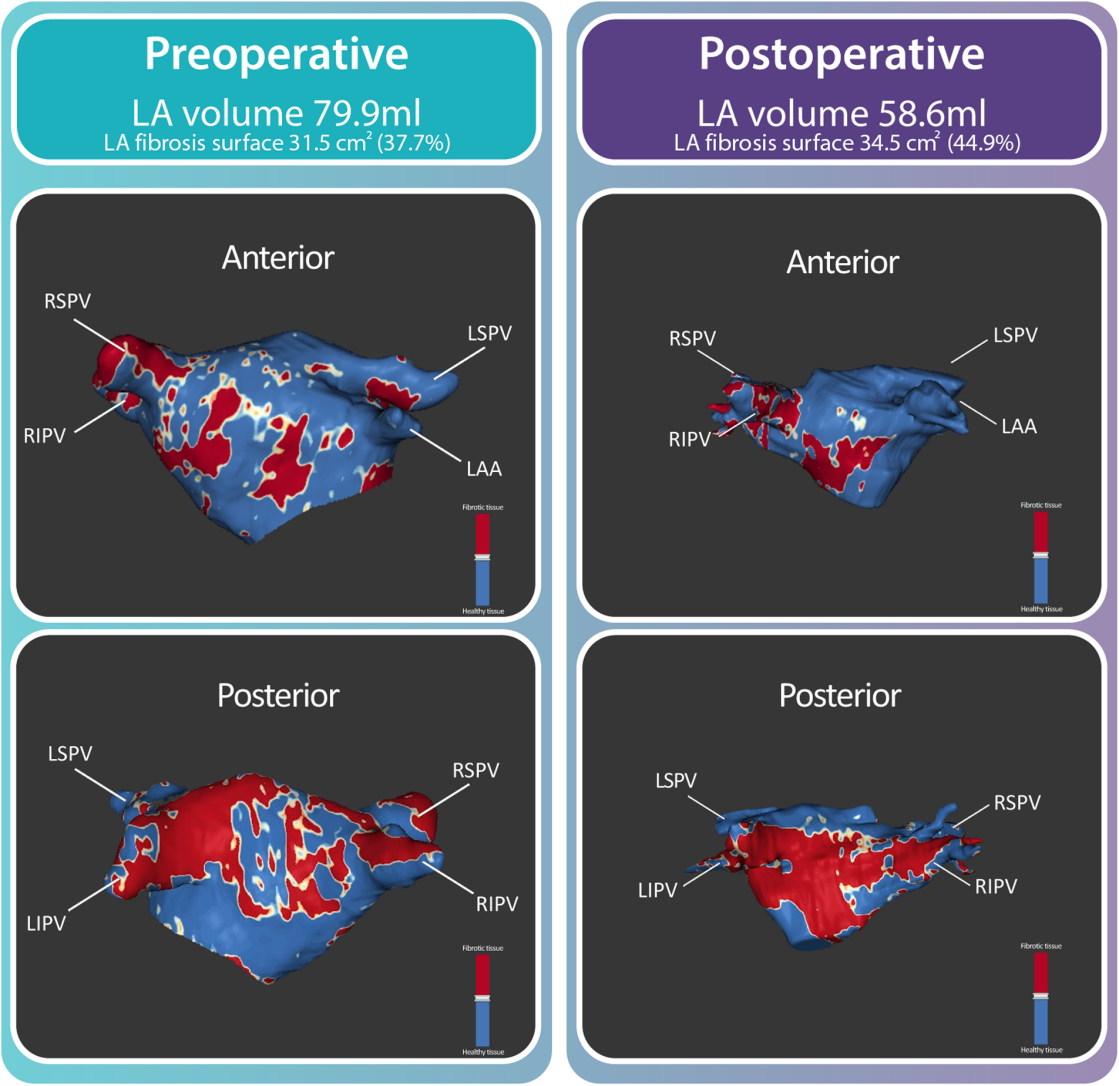
Typical example. Left atrial size and fibrotic tissue prior to and after mitral valve repair. Preoperatively, a substantial amount of fibrotic tissue was found (37.7%, 31.5 cm^2^). Postoperatively, a reduction in LA volume was observed (from 79.9 ml to 58.6 ml). This reduction of LA volume was associated with a relative increase in net LA fibrosis surface (44.9%, 34.5 cm^2^). Abbreviations: LA; left atrial, LAA; left atrial appendage, LSPV; left superior pulmonary vein, RIPV; right inferior pulmonary vein, RSPV; right superior pulmonary vein.

Analysis of the CMR images prior to surgery showed a LA volume of 79.9 ml and a total fibrosis surface of 31.5 cm^2^, covering 37.7% of the LA surface (Figure 6). Postoperatively, a reduction in LA volume was observed (from 79.9 ml to 58.6 ml). This reduction of LA volume was associated with a relative increase in net LA fibrosis surface (44.9%) while the total fibrosis area (34.5 cm^2^) increased.

## Discussion

Previous studies suggest that LA fibrosis might be present in primary MR patients (1, 2, 11, 14–17). However, most of these studies involve patients with AF, animals or comprise of myocardial fibrosis analysis with either histopathology or CMR techniques less sensitive to atrial fibrosis detection as compared to 3D LGE CMR.

Among these studies, an animal study demonstrated that LA fibrosis increases with duration of MR. (17) The longer LA volume overload was present, the more LA fibrosis was detected. The increase in amount of fibrotic tissue was not uniform among different LA areas but had a specific geometric distribution. Fibrotic tissue was predominantly found in the LA posterior wall and around the pulmonary vein orifices. The heterogeneity in distribution of LA fibrosis was found to relate to AF in this study.

To the best of our knowledge, there is no literature available on the development of LA fibrosis after mitral valve surgery. However, recent cardiology guidelines suggest to take LA (reversed) remodeling into account for clinical decision making in patients suffering from chronic severe primary MR (27). As LA fibrosis is part of this remodeling process, it might potentially have a role in this patient population and may have consequences for timing and indication of MVR surgery (27, 28, 40–42).

The ALIVE study is a single center, prospective pilot study to evaluate LA fibrosis in patients suffering from severe primary MR using CMR imaging. The primary objective is to quantify the presence, amount and distribution of LA fibrosis in primary MR patients and to determine the effect of reduced LA volume overload after MVR surgery on this specific tissue feature of atrial remodelling.

## Conclusion and implications

The ALIVE study is the first in human study for LA fibrosis characterization in primary MR patients prior to and after MVR surgery. This study may provide novel insights into the pathophysiological mechanism of cardiac (reversed) remodeling on a tissue level. The gained novel insight might potentially be clinically valuable for indication and timing of MVR surgery in patients with primary MR.

## Data Availability

The raw data supporting the conclusions of this article will be made available, without undue reservation.

## References

[1] Cameli M Lisi M Righini FM Massoni A Natali BM Focardi M Usefulness of atrial deformation analysis to predict left atrial fibrosis and endocardial thickness in patients undergoing mitral valve operations for severe mitral regurgitation secondary to mitral valve prolapse. Am J Cardiol. (2013) 111(4):595–601. 10.1016/j.amjcard.2012.10.049 23211360

[2] Zhang D Li B Li B Tang Y. Regulation of left atrial fibrosis induced by mitral regurgitation by SIRT1. Sci Rep-Uk. (2020) 10(1):7278. 10.1038/s41598-020-64308-6 PMC7190846 32350389

[3] Constant Dit Beaufils AL Huttin O Jobbe-Duval A Senage T Filippetti L Piriou N Replacement myocardial fibrosis in patients with mitral valve prolapse: relation to mitral regurgitation, ventricular remodeling, and arrhythmia. Circulation. (2021) 143(18):1763–74. 10.1161/CIRCULATIONAHA.120.050214 33706538

[4] Gao L Wang LY Liu ZQ Jiang D Wu SY Guo YQ TNAP Inhibition attenuates cardiac fibrosis induced by myocardial infarction through deactivating TGF-beta 1/smads and activating P53 signaling pathways. Cell Death Dis. (2020) 11(1):44. 10.1038/s41419-020-2243-4 PMC6976710 31969558

[5] Liew R Khairunnisa K Gu Y Tee N Yin NO Naylynn TM Role of tumor necrosis factor-alpha in the pathogenesis of atrial fibrosis and development of an arrhythmogenic substrate. Circ J. (2013) 77(5):1171–9. 10.1253/circj.CJ-12-1155 23370453

[6] Lee KN Kim DY Boo KY Kim YG Roh SY Baek YS Therapeutic implications of galectin-3 in patients with atrial fibrillation. Sci Rep. (2022) 12(1):784. 10.1038/s41598-022-04894-9 35039576 PMC8764095

[7] Weng W Choudhury R Sapp J Tang A Healey JS Nault I The role of brain natriuretic peptide in atrial fibrillation: a substudy of the substrate modification with aggressive blood pressure control for atrial fibrillation (SMAC-AF) trial. BMC Cardiovasc Disord. (2021) 21(1):445. 10.1186/s12872-021-02254-5 34530738 PMC8447763

[8] Liao CH Akazawa H Tamagawa M Ito K Yasuda N Kudo Y Cardiac mast cells cause atrial fibrillation through PDGF-A-mediated fibrosis in pressure-overloaded mouse hearts. J Clin Invest. (2010) 120(1):242–53. 10.1172/JCI39942 20038802 PMC2798688

[9] Gonzalez A Schelbert EB Diez J Butler J. Myocardial interstitial fibrosis in heart failure biological and translational perspectives. J Am Coll Cardiol. (2018) 71(15):1696–706. 10.1016/j.jacc.2018.02.021 29650126

[10] King JB Azadani PN Suksaranjit P Bress AP Witt DM Han FT Left atrial fibrosis and risk of cerebrovascular and cardiovascular events in patients with atrial fibrillation. J Am Coll Cardiol. (2017) 70(11):1311–21. 10.1016/j.jacc.2017.07.758 28882227

[11] Mandoli GE Pastore MC Benfari G Bisleri G Maccherini M Lisi G Left atrial strain as a pre-operative prognostic marker for patients with severe mitral regurgitation. Int J Cardiol. (2021) 324:139–45. 10.1016/j.ijcard.2020.09.009 32920069

[12] Chelu MG King JB Kholmovski EG Ma JJ Gal P Marashly Q Atrial fibrosis by late gadolinium enhancement magnetic resonance imaging and catheter ablation of atrial fibrillation: 5-year follow-up data. J Am Heart Assoc. (2018) 7(23):e006313. 10.1161/JAHA.117.006313 30511895 PMC6405558

[13] Dzeshka MS Lip GYH Snezhitskiy V Shantsila E. Cardiac fibrosis in patients with atrial fibrillation mechanisms and clinical implications. J Am Coll Cardiol. (2015) 66(8):943–59. 10.1016/j.jacc.2015.06.1313 26293766

[14] Wu YF Gao P Fang Q Liu YT Cheng K Cheng ZW Mitral valve regurgitation is associated with left atrial fibrosis in patients with atrial fibrillation. J Electrocardiol. (2022) 70:24–9. 10.1016/j.jelectrocard.2021.11.031 34844143

[15] Qiao Y Wu L Hou B Sun W Zheng L Ding L Functional mitral regurgitation: predictor for atrial substrate remodeling and poor ablation outcome in paroxysmal atrial fibrillation. Medicine (Baltimore). (2016) 95(30):e4333. 10.1097/MD.0000000000004333 27472715 PMC5265852

[16] Thiedemann KU Ferrans VJ. Left atrial ultrastructure in mitral valvular disease. Am J Pathol. (1977) 89(3):575–604. 145805 PMC2032253

[17] Li B Luo FL Luo XK Li B Qi L Zhang D Effects of atrial fibrosis induced by mitral regurgitation on atrial electrophysiology and susceptibility to atrial fibrillation in pigs. Cardiovasc Pathol. (2019) 40:32–40. 10.1016/j.carpath.2019.01.006 30836303

[18] Hopman L Bhagirath P Mulder MJ Eggink IN van Rossum AC Allaart CP Quantification of left atrial fibrosis by 3D late gadolinium-enhanced cardiac magnetic resonance imaging in patients with atrial fibrillation: impact of different analysis methods. Eur Heart J Cardiovasc Imaging. (2022) 23(9):1182–90. 10.1093/ehjci/jeab245 35947873 PMC9365307

[19] Kis Z Hendriks AA Muka T Bramer WM Kovacs I Szili-Torok T. The role of atrial fibrosis detected by delayed—enhancement MRI in atrial fibrillation ablation. Curr Med Imaging Rev. (2020) 16(2):135–44. 10.2174/1573405614666180806130327 32003313

[20] Marrouche NF Wazni O McGann C Greene T Dean JM Dagher L Effect of MRI-guided fibrosis ablation vs conventional catheter ablation on atrial arrhythmia recurrence in patients with persistent atrial fibrillation: the DECAAF II randomized clinical trial. JAMA. (2022) 327(23):2296–305. 10.1001/jama.2022.8831 35727277 PMC9214588

[21] Nishimura RA Vahanian A Eleid MF Mack MJ. Mitral valve disease–current management and future challenges. Lancet. (2016) 387(10025):1324–34. 10.1016/S0140-6736(16)00558-4 27025438

[22] Lazam S Vanoverschelde JL Tribouilloy C Grigioni F Suri RM Avierinos JF Twenty-Year outcome after mitral repair versus replacement for severe degenerative mitral regurgitation: analysis of a large, prospective, multicenter, international registry. Circulation. (2017) 135(5):410–22. 10.1161/CIRCULATIONAHA.116.023340 27899396

[23] Le Tourneau T Messika-Zeitoun D Russo A Detaint D Topilsky Y Mahoney DW Impact of left atrial volume on clinical outcome in organic mitral regurgitation. J Am Coll Cardiol. (2010) 56(7):570–8. 10.1016/j.jacc.2010.02.059 20688212

[24] Essayagh B Antoine C Benfari G Messika-Zeitoun D Michelena H Le Tourneau T Prognostic implications of left atrial enlargement in degenerative mitral regurgitation. J Am Coll Cardiol. (2019) 74(7):858–70. 10.1016/j.jacc.2019.06.032 31416529

[25] Stassen J van Wijngaarden AL Butcher SC Palmen M Herbots L Bax JJ Prognostic value of left atrial reservoir function in patients with severe primary mitral regurgitation undergoing mitral valve repair. Eur Heart J Cardiovasc Imaging. (2022) 24(1):142–151. 10.1093/ehjci/jeac058 PMC9762939 35301525

[26] van Wijngaarden AL Mantegazza V Hiemstra YL Volpato V van der Bijl P Pepi M Prognostic impact of extra-mitral valve cardiac involvement in patients with primary mitral regurgitation. JACC Cardiovasc Imaging. (2022) 15(6):961–70. 10.1016/j.jcmg.2021.11.009 35033499

[27] Vahanian A Beyersdorf F Praz F Milojevic M Baldus S Bauersachs J 2021 ESC/EACTS guidelines for the management of valvular heart disease. EuroIntervention. (2022) 17(14):e1126–e96. 10.4244/EIJ-E-21-00009 34931612 PMC9725093

[28] Bonow RO O'Gara PT Adams DH Badhwar V Bavaria JE Elmariah S Focused update of the 2017 ACC expert consensus decision pathway on the management of mitral regurgitation: a report of the American college of cardiology solution set oversight committee. J Am Coll Cardiol. (2020) 75(17):2236–70. 10.1016/j.jacc.2020.02.005 32068084

[29] Thomas L Abhayaratna WP. Left atrial reverse remodeling: mechanisms, evaluation, and clinical significance. JACC Cardiovasc Imaging. (2017) 10(1):65–77. 10.1016/j.jcmg.2016.11.003 28057220

[30] Jalife J Kaur K. Atrial remodeling, fibrosis, and atrial fibrillation. Trends Cardiovasc Med. (2015) 25(6):475–84. 10.1016/j.tcm.2014.12.015 25661032 PMC5658790

[31] Stassen J van Wijngaarden AL Wu HW Palmen M Tomsic A Delgado V Left atrial remodeling after mitral valve repair for primary mitral regurgitation: evolution over time and prognostic significance. J Cardiovasc Dev Dis. (2022) 9(7):230. 10.3390/jcdd9070230 35877592 PMC9320730

[32] Baumgartner H Falk V Bax JJ De Bonis M Hamm C Holm PJ 2017 ESC/EACTS guidelines for the management of valvular heart disease (vol 38, pg 2739, 2017). Eur Heart J. (2018) 39(21):1980. 10.1093/eurheartj/ehx636 29087457

[33] Spertus JA Jones PG Sandhu AT Arnold SV. Interpreting the Kansas city cardiomyopathy questionnaire in clinical trials and clinical care: jACC state-of-the-art review. J Am Coll Cardiol. (2020) 76(20):2379–90. 10.1016/j.jacc.2020.09.542 33183512

[34] Bisbal F Guiu E Calvo N Marin D Berruezo A Arbelo E Left atrial sphericity: a new method to assess atrial remodeling. Impact on the outcome of atrial fibrillation ablation. J Cardiovasc Electrophysiol. (2013) 24(7):752–9. 10.1111/jce.12116 23489827

[35] Bertelsen L Alarcon F Andreasen L Benito E Olesen MS Vejlstrup N Verification of threshold for image intensity ratio analyses of late gadolinium enhancement magnetic resonance imaging of left atrial fibrosis in 1.5 T scans. Int J Cardiovasc Imaging. (2020) 36(3):513–20. 10.1007/s10554-019-01728-0 31748945 PMC7080681

[36] Coutinho GF Antunes MJ. Mitral valve repair for degenerative mitral valve disease: surgical approach, patient selection and long-term outcomes. Heart. (2017) 103(21):1663–9. 10.1136/heartjnl-2016-311031 28566474

[37] Di Tommaso E Rapetto F Guida GA Zakkar M Bruno VD. Benefits of mitral valve repair over replacement in the elderly: a systematic review and meta-analysis. J Card Surg. (2021) 36(7):2524–30. 10.1111/jocs.15506 33783032

[38] Dilaveris P Antoniou CK Manolakou P Tsiamis E Gatzoulis K Tousoulis D. Biomarkers associated with atrial fibrosis and remodeling. Curr Med Chem. (2019) 26(5):780–802. 10.2174/0929867324666170918122502 28925871

[39] Brundel B Ai X Hills MT Kuipers MF Lip GYH de Groot NMS. Atrial fibrillation. Nat Rev Dis Primers. (2022) 8(1):21. 10.1038/s41572-022-00347-9 35393446

[40] Ling LH EnriquezSarano M Seward JB Orszulak TA Schaff HV Bailey KR Early surgery in patients with mitral regurgitation due to flail leaflets—a long-term outcome study. Circulation. (1997) 96(6):1819–25. 10.1161/01.CIR.96.6.1819 9323067

[41] Rosenhek R Rader F Klaar U Gabriel H Krejc M Kalbeck D Outcome of watchful waiting in asymptomatic severe mitral regurgitation. Circulation. (2006) 113(18):2238–44. 10.1161/CIRCULATIONAHA.105.599175 16651470

[42] Zilberszac R Heinze G Binder T Laufer G Gabriel H Rosenhek R. Long-Term outcome of active surveillance in severe but asymptomatic primary mitral regurgitation. JACC Cardiovasc Imaging. (2018) 11(9):1213–21. 10.1016/j.jcmg.2018.05.014 30031699

